# Lebanese Hospital-Based Rheumatoid Arthritis Registry: Characteristics of Patients and Comparison with Other Populations

**DOI:** 10.31138/mjr.33.2.218

**Published:** 2022-06-30

**Authors:** Houda Ziadeh, Monique Chaaya, Samar Rachidi, Khalil El Asmar, Amal Al-Hajje, Lamiaa Hamieh, Vicky Nahra, Imad Uthman

**Affiliations:** 1 Clinical and Epidemiological Research Laboratory, Faculty of Pharmacy, Doctoral School of Science and Technology, Lebanese University, Beirut, Lebanon,; 2 Department of Epidemiology and Population Health, Faculty of Health Sciences, American University of Beirut, Beirut, Lebanon,; 3 Clinical and Epidemiological Research Laboratory, Faculty of Pharmacy-Clinical Pharmacy Department, Lebanese University, Beirut, Lebanon,; 4 Department of Dermatology, American University of Beirut, Beirut, Lebanon,; 5 Division of Rheumatology, Faculty of Medicine, American University of Beirut, Beirut, Lebanon

**Keywords:** rheumatoid Arthritis, AUBMC, Lebanon, RA Registry, biologic agents

## Abstract

**Objectives:**

The aim of the Lebanese hospital-based Rheumatoid Arthritis (RA) registry, initiated in 2011, is to evaluate the safety and efficacy of biologic agents among patients seeking care at the American University of Beirut Medical Center (AUBMC). We aimed to characterize the demographic and clinical profile of RA patients included in the Lebanese registry. We compared our results with those issued from Middle Eastern and non-Middle Eastern registries.

**Methods:**

195 Patients enrolled in the RA registry from 2011 to 2018 were considered in this study. Patients enrolled in the registry were eligible to be treated with biologics, but 56 patients remained biologics naïve. Patients were reassessed every six months.

**Results:**

The highest proportion of patients were female (81%). The mean age was 55.53±15 years, and the disease duration was 11.38±7.7 years. RA was diagnosed at a mean age of 44.13±16 years. Almost one-third of RA patients were smokers (29.2%) and 15% consumed alcohol. Comorbidities included cardiac diseases (30.8%), hypertension (24.6%), hyperlipidemia (11.8%), diabetes mellitus (9.2%), and Hypothyroidism (6.2%). Three cases of cancer and seven cases of tuberculosis were reported. The mean of the Disease Activity Score (DAS28) was 3.75 ± 2.28 with no difference according to gender; the mean of the Health Assessment Questionnaire (HAQ) score showed a significant difference between females and males (1.02 ± 0.84 and 0.61 ± 0.7 respectively). Methotrexate was the most commonly used medication. There was non-significant difference in taking biologics according to gender.

**Conclusion:**

Our findings are similar to other studies in terms of gender distribution. The higher mean age at diagnosis compared to other populations could indicate a delay in seeking appropriate care. The Lebanese RA registry provides valuable data on pharmacological interventions used and an opportunity to follow up to examine the effectiveness of different therapeutics and to monitor their side effects.

## INTRODUCTION

Rheumatoid Arthritis (RA) is a chronic inflammatory disease, characterised by symmetric polyarthritis with progressive joint damage^1^ which can lead to significant disability and functional loss. It is also a burden on the individual, the health care system, and society. In most countries, the prevalence in the adult population is approximately 0.5–1.0%.^2,3^ The age at onset is between 40 and 60 years. Women represent 70 % of patients with RA.^4^ In Lebanon, a COPCORD study conducted in 2012 reported a prevalence of 1%.^5^

In late 1990, the introduction of biologics represented a significant advance in the field of Rheumatology,^6^ and transformed what was once considered for many a devastating and incurable disease, into one that can be mostly controlled.^7^

Biological agents are used by an increasing number of RA patients, although their cost is high. However, their effectiveness and safety still need to be established; that is why registries were launched.

Registries have become an essential complement to data obtained from randomised clinical trials (RCTs) which could not show conclusive results about safety and therapeutic strategies due to limited duration of exposure to drugs and a limited number of patients involved.^8^ Registries can be drug-based if the patients enrolled are starting a specific medication, or disease-based if the patients enrolled have a particular disease or both. The majority of registries allow an evaluation of outcomes referring to a comparator group of RA patients.^9^

Clinical registries provide information on drug safety and drug-related outcomes, in addition to generating adequate data sets for research questions and a powerful research database. Thus, registries are used to provide evidence on the long-term efficacy of therapies and to establish a powerful research database. Clinical Rheumatology registries have been established in many European countries: The British Society for Rheumatology Biologics Registry (BSRBR)^10^; Dutch Rheumatoid Arthritis Monitoring (DREAM) registry^11^; German RABBIT registry^12^; Finnish (ROB-FIN) registry of rheumatic diseases^13^; Norwegian (NOR-DMARD) registry^14^; Spanish registry (BIOBADASER)^15^; Swedish RA registry (ARTIS)^16^; These registries have resulted in many landmark articles that have redefined our practice in the field of Rheumatology.

In the Middle East, few RA registries have been recently established. In 2014, the Dubai Health Authority (DHA) launched the Dubai Arthritis Registry (DAR)^17^ which records the prevalence of six major musculoskeletal diseases including Rheumatoid Arthritis. The Kuwait Registry for Rheumatic Diseases (KRRD)^18^ electronically connects patients from major hospitals in Kuwait, and currently includes more than 1600 patients with more than 8000 hospital visits.

In Lebanon, a registry of RA patients seen at the American University of Beirut Medical Centre (AUBMC) was initiated in 2011, the first of its kind in the Middle East. This registry was launched to: (1) describe the clinical and serologic characteristics of RA patients receiving biologic therapy at the American University of Beirut Medical Centre; (2) to monitor the safety signals that might ensue with the short- and long-term use of biologic therapy, and (3), to assess the efficacy of the new biologic targeted therapeutic options and establish their role in the treatment algorithms of RA.

This study describes the most important epidemiological and clinical features of patients with RA from the AUBMC RA Registry. Moreover, we aim to describe the prevalence of common comorbidities and compare our results to those from non-Middle Eastern studies. This evaluation will raise questions for future studies and provide information that could be valuable in improving care for RA patients in Lebanon and the Middle East.

## PATIENTS AND METHODS

The American University of Beirut Medical Centre (AUBMC) is located in Beirut, Lebanon, established more than 120 years ago. Its mission is to improve the health of the Lebanese community and the region. It offers exceptional quality of care to patients and leadership in innovative research and education. In 2011, a registry for Rheumatoid Arthritis patients seen at the AUBMC was initiated.

From February 2011 until April 2018, all Rheumatoid Arthritis patients eligible to take biologic therapy were approached to be included in the registry.

### Inclusion Criteria

RA Patients (18 years and above) and according to ACR criteria, were first clinically assessed by rheumatologists at AUBMC to determine if they required biologic therapy. Patients who qualified for biological treatment but refused to take it for any reason were recruited as controls. If the patient is found to fit the inclusion criteria, they are approached, and informed consent is obtained.

### Exclusion Criteria

Contraindication to the biologic agent according to the local labelling of the drug in question.

After the approval of the Institutional Review Board of the American University of Beirut, data were collected from the patient and the rheumatologist, using two forms: Rheumatologist baseline questionnaire and the Patient Baseline questionnaire.

The baseline data included socio-demographic characteristics (age, gender, working, and educational status), and clinical and health characteristics. Clinical characteristics included age at diagnosis of RA, current therapy, previous drug history, disease activity, and health status in general assessed by patients scores on DAS28 and HAQ scales respectively; other health-related variables assessed were: co-morbidities (Hypertension, Angina, Heart attack, Stroke, Epilepsy, Asthma, Chronic Obstructive Pulmonary Disease, Peptic Ulcer, Liver disease, Renal Disease, Tuberculosis, Demyelination, Diabetes, Hyperthyroidism, Depression, Cancer, Osteoporosis, Anxiety), smoking status, alcohol use, height, weight, and blood pressure.

Patients enrolled were followed up for at least six months after their first visit (by phone or in the clinic), and a Rheumatologist follow up questionnaire and a Patient follow up questionnaire was filled as well. Follow-up data included: Changes in treatment protocol (drugs, doses), a physical examination, disease activity, and general health using DAS28 and HAQ questionnaires. “Event of Special Interest Report” was filled for several adverse events if they were reported during the follow-up period. Events of special Interest include aplastic anaemia, congestive heart failure, cerebrum-vascular event, demyelinating disease, optic neuritis, malignancy, lymphoproliferative malignancy, acute coronary syndrome, pulmonary embolism, pregnancy, serious infection, tuberculosis, immunologic/infusion reaction.

All collected data were entered into a database designed using MS Access, where the user interface was designed using Borland Delphi 7. SPSS (Statistical Package for Social Sciences) for Windows, Release 21, was used to perform all statistical analyses for this study. Descriptive data analysis was performed by computing means, standard deviations, and frequency distributions for all variables as appropriate. Comparisons of categorical baseline characteristics in the registry were performed using Pearson’s chi-square test.

## RESULTS

### Socio-demographic profile

A total of 195 patients were enrolled in the registry from February 2011 until April 2018. The majority were females (81%). The mean age was 55.5± 15 years, ranging between 18 and 88 years.

The highest proportion of patients resided in Beirut Governorate (41%) followed by those from Mount Lebanon (21.5%). Less than one-tenth (8.7%) were non-Lebanese mostly from Iraq and Syria.

Data on education were available for 138 patients, the highest proportion had a college education (38%), and only seven RA patients (5%) were illiterate.

One-third of the RA patients were working at the time of the recruitment (33% full-time and 15% part-time paid employment), 25% were housewives and 10 % reported not being able to work because of a disability (**Table 1**)*.*

**Table 1.** Demographic characteristics of RA patients enrolled in the Lebanese Registry.

**Characteristic**	N*	**%**

Female	158	81

Age (mean ± SD)	195	55.53 ± 15 years

Residency		
Beirut Governorate	61	40.9
Mount Lebanon	32	21.5
Governorate	18	12.1
South Governorate	13	8.7
NON-Lebanese	12	8.1
Beqaa Governorate	8	5.4
North Governorate	3	2
Nabatieh Governorate	2	1.3
Baalbek-Hermel	149	
Governorate		
Total		

Educational Level		
University	53	38.4
Elementary	51	37
Secondary	27	19.6
Illiterate	7	5.1
Total	138	

Working status		
Working full time (paid employment)	63	33.2
	49	25.8
Working full time in the house	29	15.3
	20	10.5
Working part-time (paid employment)	14	7.4
	10	5.3
Not working due to ill health/disability	5	2.6
	190	
Retired		
Unemployed but seeking work		
Student		
Total		

* Totals less than 195 reflect missing data

### Clinical profile

The diagnosis of the disease was made at a mean age of 44.1± 16 years, ranging between 18 and 82 years. The mean disease duration was 11.3± 7 years.

To check if RA patients enrolled in the Lebanese registry had a controlled disease at baseline, we calculated the mean of DAS28 score which showed an overall average of 3.75 ± 2.28, which indicates moderate disease activity with no significant difference between males and females. The overall mean HAQ score was: 0.94 ± 0.83. A significant difference (p=0.022) was noted according to gender where females had a higher score (Table 2).

**Table 2.** Distribution of RA patients by health, clinical characteristics at baseline, and gnder.

	**Characteristic**	**N (%)**	**Females**	**Males**	**P-value**
**Health profile**	*Comorbidities*				
	Cardiac diseases	60 (30.8%)	45 (28%)	15 (40.5%)	0.15
	Hypertension	48 (24.6%)	38 (24%)	10 (27%)	0.7
	Dyslipidaemia	23 (11.8%)	15 (9.5%)	8 (21.6%)	0.04
	Diabetes	18 (9.2%)	10 (6.3%)	8 (21.6%)	0.004
	Depression	14 (7.2%)	13 (8.2%)	1 (2.7%)	0.24
	Hypothyroidism	12 (6.2%)	10 (6.3%)	2 (5.4%)	0.83
	Smoking	57 (29.2%)	40 (25.3%)	17 (45.9%)	0.013
	Alcohol consumption	30 (15.4%)	18 (11.4%)	12 (32.4%)	0.001
**Clinical Profile**	Age at diagnosis (mean + SD)	44.1 ± 16	44.78+15.9	40.79+16.8	0.22
	Years since diagnosis (mean + SD)	11.3 ± 7	10.54 + 7	15.6 + 9.6	0.001
	DAS28 Score (mean + SD)		3.82 + 2.27	3.4 + 2.3	0.42
	DAS 28 categorized				
	Disease remission (< 2.6)	46 (26.4%)	36 (25.5%)	10 (30.3%)	
	Low disease activity (2.6 – 3.2)	8 (4.6%)	5 (3.5%)	3 (9.1%)	
	Moderate disease activity (3.2 – 5.1)	64 (36.8%)	52 (36.9%)	12 (36.4%)	
	Severe disease activity (> 5.1)	56 (32.2%)	48 (34%)	8 (24.2%)	
	*HAQ Score*		1.02 + 0.84	0.61 + 0.7	0.022
	HAQ categorized				
	Mild to moderate disability (0–1)	110 (56.4%)	82 (51.9%)	28 (75.7%)	
	Moderate to severe disability (1–2)	63 (32.3%)	55 (34.8%)	8 (21.6%)	
	Severe to very severe disability (2–3)	22 (11.3%)	21 (13.3%)	1 (2.7%)	
	*Treatment at baseline*				
	MTX	89 (45.6%)	74 (46.8%)	15 (40.5%)	0.48
	DMARDs	31 (15.9%)	25 (15.8%)	6 (16.2%)	0.95
	Biologics	18 (9.2%)	14 (8.9%)	4 (10.8%)	0.71

Regarding treatment at baseline, methotrexate (MTX) was the most commonly used medication (45.6%) followed by the other c-DMARDs (16%) and biological agents (9.2%).

Several patients were taking a combination of two treatments (biologics and DMARDs, or biologics and MTX), but none was taking a combination of the three at the same time. There was non-significant difference in taking biologics according to gender.

No significant difference in DAS28 and HAQ scores were found between those who took biologics and those who did not. A weak correlation (*r*=0.2) with p value 0.004 was observed only between the age of RA patients in the registry and the HAQ score.

### Health Profile

Cardiac diseases (angina, heart attack and stroke), was the most common comorbid condition reported at baseline (31%), followed by hypertension (24.6%), dyslipidaemia (12%), and then diabetes (9%). Seven cases of tuberculosis (TB) (among which 2 were active cases) were reported at the enrolment.

**Figure 1.**
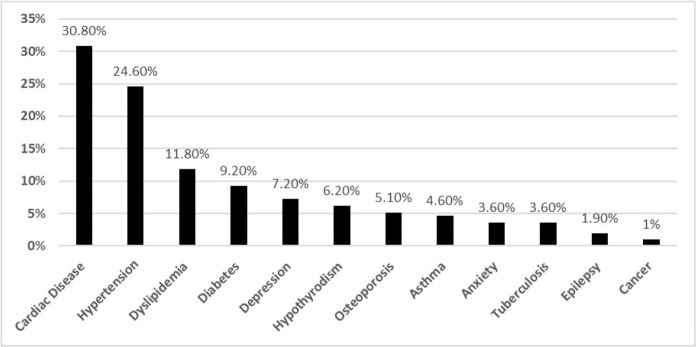
Prevalence of comorbidities in RA patients.

The prevalence of comorbidities in RA patients enrolled in the registry is shown in **Figure 1***.* Only diabetes and dyslipidaemia showed a statistically significant difference according to gender*.*

Smokers represented 29% of the RA registry population. Fifteen percent of RA patients consumed alcohol. **Table 2** shows the distribution of RA patients by health, clinical characteristics at baseline, and gender.

## DISCUSSION

Clinical registries for RA have shown their value in describing patients’ profiles and in capturing the benefit of long-term therapies routinely used in the management of the disease. In this paper, we presented demographic characteristics, comorbid diseases, disease activity, and treatment at baseline of 195 RA patients in the AUBMC Lebanese registry, one of the few registries in the region and the only one in Lebanon.

We tabulated all the characteristics of RA patients treated at AUBMC over 7 years (from February 2011 until April 2018) by gender and compared them to patients enrolled in registries in GCC countries, Europe, and the US. Some of the characteristics were found to be unique to our patients.

RA patients in the registry were female in the majority, a finding consistent with what has been described in the UAE (80%)^19^ and Jordan (83.5%).^20^ The mean age of RA patients in the registry was also similar to that reported worldwide (56 ± 13 years)^21^ but higher than that reported for the RA registry patients in Kuwait (50.6 ± 12 years)^18^ and UAE,^19^ and significantly lower than that in USA (CORRONA) (60 ± 13 years)^9^ and some countries in Europe.^9^

The mean disease duration in our study at enrolment (11.3 ± 7 years) is higher than that observed in Kuwait (6.1 years),^18^ UAE (6.1 years),19 and Saudi cohorts (5.51 years).^22^ This could indicate that we do not have a delay in RA diagnosis and in the presentation to a rheumatologist doctor. Ongoing awareness campaigns, in addition to the primary role of the Lebanese Rheumatologists on keeping up update with the development of new RA treatments and the implementation of new guidelines, had played a positive role in the prolongation of the disease duration.

Enrolment of non-Lebanese patients in the registry, especially from Iraqi and Syria may be due to the proximity of these two countries and the reputation of AUBMC as the main referral centre for Lebanon and the region.

The prevalence of some behavioural risk factors such as smoking and alcohol use reflect their high rates in the Lebanese population which is one of the highest with regards to smoking in the region, especially among women.^23^

Concerning comorbidities, cardiac diseases, hypertension, dyslipidaemia, and diabetes were the most prevalent medical conditions among the RA registry patients. In Kuwait, diabetes mellitus, hypertension, and bronchial asthma (20.8%, 20.2%, and 11.7% respectively) were the most reported comorbidities among patients enrolled in the Kuwait Registry. In Qatar, hypertension, diabetes mellitus, and dyslipidaemia (24.2%, 20.6%, and 10.9% respectively) were the most reported among RA patients in the Qatar Rheumatoid Arthritis Registry.^24^ This difference in comorbidities between Lebanon and Gulf countries may be related to the differences in living conditions and dietary habits, and attributed to genetic factors as well.

The prevalence of diabetes is close to the national prevalence figure reported by the International Diabetes Federation (2015). A higher prevalence of DM is reported in RA patients of UAE and Kuwait registries (34.5 % and 20.8% respectively). The high prevalence of diabetes and its difference from that in Lebanon is not surprising since, in 2013, Kuwait was ranked ninth in the top 10 countries with a high prevalence in DM.^25^

The high prevalence of hypertension in RA patients in the registries (Lebanese Registry, Qatar Registry and KRRD) reflects the high prevalence of hypertension in the Arab region (29.5%).^26^

The bronchial asthma is ranked third in the common comorbidities in RA patients in the Kuwait Registry (11.5%) but ranked 8^th^ in the common comorbidities in the Lebanese Registry (4.5%). The differences concerning asthma between the 2 registries are expected given the high prevalence of this disease in the Kuwait’s population (15%)^27^ and its low prevalence in Lebanon (9%).^28^

The frequency of TB infection in the Lebanese Registry is relatively low compared to UAE (12.6%)^19^ which may be due to the sample size.

As for the treatment at baseline, the highest percentage of prescriptions was for MTX reflecting the implementation and acceptance of global guidelines by the rheumatologists at AUBMC.^29^

The computing DAS28 score indicates that RA patients were far away from remission, and also, more than half of them had moderate to severe disease activity. This finding may suggest that the medical treatment at baseline was insufficient. Although the disease activity was high in most patients, it was surprising that disability (referring to HAQ score) was not at an advanced level. In Jordan, 51% of RA patients had high disease activity and only 5% were in remission^30^ (comparing to 32% and 26% respectively in the Lebanese RA Registry). This difference may be explained by the better access to medical care in Lebanon and the delayed disease presentation to a rheumatologist in Jordan.

## CONCLUSION

Rheumatoid Arthritis is known to be a heterogeneous disease with various characteristics and clinical presentation. This is the first study about the profile of Rheumatoid Arthritis patients extracted from the first registry of RA patients in Lebanon launched by the AUBMC in 2011.

Results obtained concerning the high prevalence of smoking among RA patients in the Lebanese registry highlighted the importance of raising awareness about the negative effect of this habit on overall health in general and on Rheumatoid Arthritis in particular. Moreover, given that the majority of RA patients enrolled in the Lebanese Registry had moderate to severe disease activity, the improvement of “Treat To Target” strategy is important to reach remission or low disease activity among RA patients in Lebanon.

Further analyses will be done and reported in an upcoming paper in order (1) to compare between patients who took biological agents and those who did not, in terms of prognosis referring to the Health Assessment Questionnaire (HAQ) and the Disease Activity Score (DAS28), and (2) to compare safety in terms of adverse effects and comorbidities between those exposed and non-exposed to biologics therapies.

Efforts in the implementation of this first RA registry, launched at the AUBMC, in other university hospitals should be considered, in order to have valuable epidemiological and clinical data in addition to risk group monitoring concerning the Rheumatoid Arthritis disease in Lebanon.
